# Understanding Inflammatory Responses in the Manifestation of Prothrombotic Phenotypes

**DOI:** 10.3389/fcell.2020.00073

**Published:** 2020-02-14

**Authors:** Shankar Chanchal, Aastha Mishra, Manvendra Kumar Singh, Mohammad Zahid Ashraf

**Affiliations:** ^1^Department of Biotechnology, Faculty of Natural Sciences, Jamia Millia Islamia, New Delhi, India; ^2^Signature Research Program in Cardiovascular and Metabolic Disorders, Duke-NUS Graduate Medical School Singapore, National Heart Centre Singapore, Singapore, Singapore

**Keywords:** sterile inflammation, inflammasome, thrombosis, endothelial and platelet activation, immune cell activation

## Abstract

Inflammasome complex is a multimeric protein comprising of upstream sensor protein of nucleotide-binding oligomerization domain (NOD)-like receptor family. It has an adaptor protein apoptosis-associated speck-like protein and downstream effector cysteine protease procaspase-1. Activation of inflammasome complex is body’s innate response to pathogen attack but its abnormal activation results in many inflammatory and cardiovascular disorders including thrombosis. It has displayed a prominent role in the clot formation advocating an interplay between inflammation and coagulation cascades. Therefore, elucidation of inflammasome and its molecular mechanisms in the manifestation of prothrombotic phenotypes becomes pertinent. Thrombosis is the formation and propagation of blood clot in the arterial or venous system due to several interactions of vascular and immune factors. It is a prevalent pathology underlying disorders like venous thromboembolism, stroke and acute coronary syndrome; thus, making thrombosis, a major contributor to the global disease burden. Recently studies have established a strong connection of inflammatory processes with this blood coagulation disorder. The hemostatic balance in thrombosis gets altered by the inflammatory mechanisms resulting in endothelial and platelet activation that subsequently increases secretion of several prothrombotic and antifibrinolytic factors. The upregulation of these factors is the critical event in the pathogenesis of thrombosis. Among various inflammasome, nucleotide-binding domain, leucine-rich-containing family, pyrin domain containing 3 (NLRP3) is one of the best-studied sterile inflammasome strengthening a link between inflammation and coagulation in thrombosis. NLRP3 activation results in the catalytic conversion of procaspase-1 to active caspase-1, which facilitate the maturation of interleukin-1β (IL-1β) and interleukin-18. These cytokines are responsible for immune cells activation critical for immune responses. These responses further results in endothelial and platelet activation and aggregation. However, the exact molecular mechanism related to the pathogenesis of thrombosis is still elusive. There have been several reports that demonstrate Tissue factor (TF)-mediated signaling in the production of pro-inflammatory cytokines enhancing inflammation by activating protease-activated receptors on various cells, which lead to additional cytokine expression. Therefore, it would be illuminating to interpret the inflammasomes regulation in coagulation and inflammation. This review, thus, tries to comprehensively compile emerging regulatory roles of the inflammasomes in thrombosis and discusses their molecular pathways in the manifestation of thrombotic phenotypes.

## Introduction

Inflammasome complex is a multimeric protein comprising of upstream sensor protein, adaptor protein and the cysteine protease procaspase-1 (Schroder and Tschopp, 2010). The sensor includes NLRs [Nucleotide-binding oligomerization domain (NOD) and leucine-rich repeat (LRR)-containing receptors], AIM2 (Absent in melanoma-2) or pyrin and the adaptor molecule ASC [an apoptosis-associated speck-like protein containing a caspase activation and recruitment domain (CARD)]. Most members of the NLRs family have a tripartite structure such as carboxyl-terminal LRR domain, which is involved in stimuli recognition. It recognizes infectious pathogen-associated molecular patterns (PAMPs) and endogenous damage-associated molecular patterns (DAMPs) (Chen and Nuñez, 2010). With the exposure to stimuli, inflammasome forms a complex. Procaspase-1 is recruited by ASC into the complex that converts it into its active form, caspase-1 autocatalytically. Thus, caspase-1 converts pro interleukin-1β (IL-1β) and IL-18 into their active forms leading to inflammation (Malik and Kanneganti, 2017). The DAMP associated activation of inflammation leads to the responses termed as sterile inflammation. These responses induce various immune cells such as dendritic cells (DCs) and macrophages that lead to inflammation (Shen et al., 2013). Among several inflammasomes, the most characterized are NLR members NLRP1 (Boyden and Dietrich, 2006), NLRP3 (Martinon et al., 2006), NLRC4 (Zhao et al., 2011) as well as non-NLR inflammasome, AIM-2 like receptor member (Fernandes-Alnemri et al., 2009). Slight differences among the NLR members are based on their structure. For example, NLRP1 contains both pyrin and CARD domains, resulting in the ability to recruit procaspase 1 with or without ASC (Proell et al., 2008). Out of these four, nucleotide-binding domain, leucine-rich-containing family, pyrin domain containing 3 (NLRP3) inflammasome, also known as cryopyrin is the best investigated inflammasome encoded by NLRP3 gene located on chromosome 1. It takes part in both sterile as well as non-sterile inflammation and is expressed in innate immune cells like macrophages, monocytes, DCs, neutrophils, lymphocytes, epithelial cells, endothelial cells (ECs), and osteoclasts (Kummer et al., 2007; He et al., 2016). Though NLRP3 is activated in response to the widest range of stimuli but its precise mechanism of activation continues to be arguable (Guo et al., 2015). Furthermore, few upstream mechanisms have been suggested for NLRP3 inflammasome activation. These includes interaction of pannexin1 and P2X purinergic receptor 7 (P2 × 7) ATP gated ion channel (Silverman et al., 2009), phagolysosomal destabilization and mitochondrial ROS production (Lamkanfi and Dixit, 2014 and Vanaja et al., 2015). However, in some cells such as monocytes, macrophages and DCs, stimuli do not activate NLRP3 directly. Activation rather requires a pre-treatment, also known as priming, with microbial stimuli, cytokines or endogenous molecules (He et al., 2016). On the other hand, NLRP3 found in platelets does not require any pre-treatment as NLRP3 and its components are constitutively expressed in them (Murthy et al., 2017).

Recently inflammasome have been implicated in the development of thromboembolic disorders (Gupta et al., 2017; Yadav et al., 2019). This could be arisen from the fact that inflammation and hemostasis are the two highly interrelated processes that acts in concert with each other functioning in a positive feedback loop (Foley and Conway, 2016). Any disturbance or loss in the control of these systems results in a diseased state or mutual amplification contributing to the onset of disease. Thrombus formation is one such example of the contribution of the interdependent interaction of these pathological processes. Inflammation plays a major role in the thrombus formation via activation of the coagulation system (Foley and Conway, 2016). Inflammation induces coagulation while coagulation amplifies inflammation (Margetic, 2012).

Thrombosis is the formation and propagation of blood clot inside blood vessels due to interactions of several blood, vascular and immune factors. It generally occurs when there is a disturbance in the balance between clot formation and its dissolution. Arterial and venous thrombosis though have separate manifestations but their pathophysiology is quite similar (Koupenova et al., 2016). Arterial (white) thrombus is rich in platelet and forms at places of high shear flow while venous (red) thrombosis is rich in fibrin and red blood cells and forms at places of slow shear flow (Koupenova et al., 2016); both remain multifactorial. Though Virchow’s triad describing hypercoagulability, hemodynamic changes and endothelial dysfunction contributes to the thrombus formation along with plethora of other independent risk factors such as infection (Esmon, 2009). Lately, exposure to high-altitude (HA) has also been associated with the increased frequency of occurrence and probability of thromboembolic complications (Prabhakar et al., 2019). Perhaps the extreme condition at HA such as severe dehydration, hemoconcentration, hypobaric hypoxia and low temperature would affirm the happening of these events (Gupta and Ashraf, 2012). One of our study demonstrated a direct association between NLRP3 and hypoxia-inducible factor 1-alpha (HIF-1α) in potentiating thrombosis under hypoxic conditions (Gupta et al., 2017). We demonstrated inflammation precedes coagulation in thrombosis and a concomitant increase in the relative expression of NLRP3, caspase-1, IL-1β, and IL-18 transcripts in thrombotic patients. Additionally, Yadav et al. (2019) showed activation of NLRP3 inflammasome and IL-1β release in their CD39-deficient mice resulting in thrombus formation under normal oxygen concentration. CD39 haploinsufficiency in mice shows increased expression of tissue factor (TF), fibrin and H3 histone along with neutrophil extracellular traps (NET) formation and leukocytes recruitment. All of these processes pertaining to NLRP3 described in the two studies subsequently result in the creation of prothrombotic milieu irrespective of the oxygen concentrations. Therefore, in view of the emerging regulatory roles of the inflammasome in thrombosis, this review article discusses the molecular mechanisms and signaling pathways related to inflammasome in the manifestation of thrombotic phenotypes.

## Inflammasomes in Immune Cells Activation

Stimulation of inflammasome by PAMPs and DAMPs triggers proinflammatory and antimicrobial events activating both innate and adaptive immune responses (Acosta-Rodriguez et al., 2007; Martinon et al., 2009; Blom and Poulsen, 2012). Inflammasome is a critical component of the innate immune system that mediates autocatalytic activation of caspase-1 leading to maturation and secretion of proinflammatory cytokines such as IL-1β and IL-18. These are critical for functions of DCs and macrophages in response to various microbial infection and cellular damages (Franchi et al., 2012). In addition, caspases enzymatically cleave gasdermin D to induce pyroptosis (Shi et al., 2015). On the other hand, the exact mechanism through which inflammasome influences adaptive immunity is still revealing. The adaptive immune responses through activation of toll like receptors (TLRs) via activation of AP-1/NFKB family is mainly explored. However, the influence of inflammasome-mediated IL-1 family cytokines on differentiated lymphocytes of both innate and adaptive classes recommends a significant role of inflammasomes family in adaptive immunity (Iwasaki and Medzhitov, 2015). As a response to cytokines, the maturation of antigen presenting cells such as DCs is a crucial event for T-cell mediated adaptive immune responses. This could be achieved through increased lysosomal activity that facilitates loading of microbes derived peptides onto major histocompatability complex for antigen presentation to T cells. Increased expression of co-stimulatory molecules such as CD80, CD86 and upregulation of cytokines are other pathways required for immune responses (Evavold and Kagan, 2018). Interestingly, the role of immune cells such as mast cells (MCs) and leukocytes in endothelial and platelets activation is already known (Budnik and Brill, 2018). Histamine, one of the major secretion from MCs has a strong prothrombotic effect by inducing release of von Willebrand factor (VWF) and P-selectin from Weibel palade bodies (WPBs) (Erent et al., 2007).

Furthermore, the involvement of inflammasomes in the pathophysiology of several inflammatory disorders such as systemic lupus erythematous (SLE) and rheumatoid arthritis (RA) with prominent pro-thrombotic phenotypic features is a strong link of interaction of inflammation and coagulation pathways. Table 1 outlines several such inflammatory disorders with thrombotic features that have demonstrated the involvement of inflammasomes in their pathophysiology. Inflammasome activation has been demonstrated in the Patients with SLE, which qualifies to be an independent risk factor for thrombosis. The type I interferons (IFNs), an established mediators of SLE pathogenesis has shown to be regulators of the inflammasome through identified interferon regulatory factor 1 (Liu J. et al., 2017). Similarly, Behcet disease (BD), an amalgamation between autoimmune and autoinflammatory syndromes that has a strong thrombotic component have shown the involvement of NLRP3 in increased IL-1β secretions in BD patients when compared to healthy volunteers (Kim et al., 2015). Likewise, a recent work demonstrated the enhanced expression of NOD2, NLRP3, and NLRC5 in anti-neutrophil cytoplasmic antibody (ANCA) associated Vasculitis (AAV) when compared to normal controls (Wang et al., 2019). Atherothrombotic development in AAV is a consequence of interactions between endothelial cell (ECs) and neutrophils activated by tumor necrosis factor-α (Springer and Forte, 2013). Next, the pathogenic role of NLRP3 in inflammatory bowel disease (IBD) has also been demonstrated (Liu L. et al., 2017). Patients with IBD have as much as three-fold increased risk of thromboembolic complications associated with a higher morbidity and mortality (Giannotta et al., 2015; Andrade et al., 2018).

**TABLE 1:** Thrombosis associated inflammatory disorders with the involvement of inflammasomes in their pathophysiology.

Inflammatory disorders	Thrombosis pathophysiology	Inflammasomes involvement
Behcet disease	Mostly venous thrombosis. Its manifestation is due to endothelial injury (Koc et al., 1992; Seyahi and Yurdakul, 2011).	Increased expression of NLRP3 and IL-1β (Kim et al., 2015).
Anti-neutrophil cytoplasmic antibody associated vasculitis	Mostly venous thrombosis. Cytokines such as IL-1β and TNF-α increased the expression of endothelial TF causing thrombosis (Stassen et al., 2008).	Increased expression of NOD2, NLRP3, NLRC5 inflammasome, and IL-1β, IL-18 (Wang et al., 2019).
Inflammatory bowel disease	Both (arterial and venous types). Alteration in coagulation enzymes/factor such as prothrombin, FV, FVII, FVIII, X leading to hyper-coagulatibility. Hyperhomocysteinemia (Jackson et al., 1997; Giannotta et al., 2015).	Increased expression of NLRP3 and secretion of IL-1β. Downregulation of NLRP6, NLRP12 (Mao et al., 2018)
Rheumatoid arthritis	Both (arterial and venous types). Due to endothelial injury and hypercoagubility. Hyperhomocysteinemia (Mameli et al., 2009).	Increased expression of NLRP3 and its downstream molecules (Ruscitti et al., 2015). Mutation in NLRP1 is associated with RA (Grandemange et al., 2017).
Systemic lupus erythematosus	Both (arterial and venous types). Due to endothelial injury. Increased expression of ICAM, VEGF, vWF, and VCAM (Burgos and Alarcón, 2009).	Increased expression of NLRP3, caspase 1 and IL-18 via TLR dependent NF-κB activation. AIM2 is activated with high disease activity (Ji et al., 2016; Shen et al., 2018). Polymorphism in NLRP1 is associated with the disease (Pontillo et al., 2012).
Antiphosphospholipid antibody syndrome	Both (arterial and venous types). APL antibodies induce thrombosis mediated through TF and TXA2 after endothelial and monocytes activation (Pierangeli et al., 2007; Irastorza et al., 2010).	Activation of NLRP3 inflammasome and increased caspase-1 and IL-1β production (Muller-calleja et al., 2015)
Familial mediterranean fever	Both (Arterial and venous types). Due to endothelial injury and endothelial cell dysfunction (Aksu et al., 2007; Demirel et al., 2008).	Activation of NLRP3 inflammasome and uncontrolled production of IL-1β (Migita et al., 2018)

## Inflammasome in Endothelial Activation

Vascular endothelium plays a critical role in regulating homeostasis. Under normal condition, endothelium maintains vasodilatory and local fibrinolytic state. It aids in the suppression of inflammation, leukocytes activation, platelet activation and aggregation. ECs produce thrombomodulin that activates protein C, which subsequently inactivates thrombin and hence promotes anticoagulant mechanisms. It activates tissue plasminogen activator to maintain the fibrinolytic activity through TF pathway inhibitor (TFPI). It also expresses heparin sulfate and dermatan sulfate, which stimulate antithrombin III and heparin cofactor activity that inhibits coagulation. Apart from these, endothelium produces nitric oxide (NO) and prostacyclin that maintains vasodilation. This integrity of the endothelium makes it antithrombotic in nature (Petäjä, 2011; van Hinsbergh, 2012). However, under certain stress conditions such as hypoxia upregulated ROS production, which activates endothelium changing its phenotype from antithrombotic to prothrombotic and antifibrinolytic. The activation/dysfunction of endothelium induces the expression of adhesion molecules and receptor resulting in the recruitment of leukocytes and extravasation (van Hinsbergh, 2012; Incalza et al., 2018). Endothelial activation leads the production of WPBs, which fuse with the plasma membrane and release its constituents like vWF and P-selectin inducing aggregation of platelets, monocytes and macrophages on the walls of the vasculature (Erent et al., 2007). Apart from these, ECs release endothelin and platelet-activating factor, which contributes toward vasoconstriction and platelet activation. The interaction of ECs with platelets, leukocytes and pro-inflammatory mediators enhance blood coagulation by the increased expression of TF (May et al., 2007; Yau et al., 2015). Endothelial P2Y receptors mediates TF expression through mechanisms involving Src/Fra-1 and Rho/JNK pathways (Ding et al., 2011; Liu et al., 2016). A major procoagulant molecule, TF, plays a critical role by generating coagulation proteases like thrombin and stimulating protease-activated receptors. Additionally, TF contributes to a variety of biological processes like inflammation, thrombosis, angiogenesis, cell migration and metastasis (Mackman, 2004).

**FIGURE 1:**
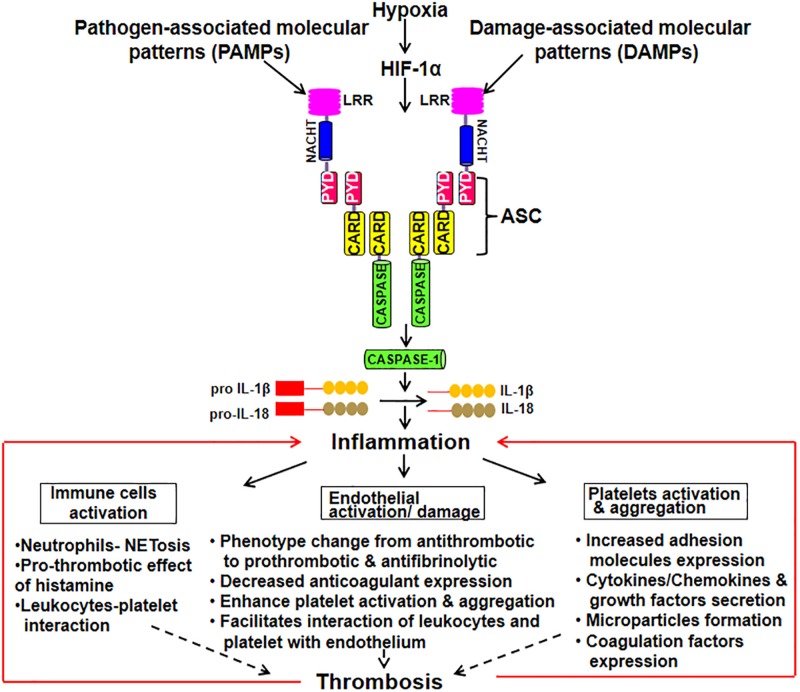
Schematic depiction of inflammasome activation leading to the manifestation of thrombotic phenotypes. Inflammasome complex is a multimeric protein comprising of upstream sensor protein, an adaptor protein, and the cysteine protease procaspase-1. The sensor includes NLRs [Nucleotide-binding oligomerization domain (NOD) and leucine-rich repeat (LRR)-containing receptors] and the adaptor molecule ASC [an apoptosis-associated speck-like protein containing a caspase activation and recruitment domain (CARD)]. Upon exposure to DAMPs and PAMPs, inflammasome forms a complex. Procaspase-1 is recruited by ASC into the complex that converts it into its active form. Thus, caspase converts pro IL-1β and IL-18 into their active forms leading to inflammation. This, subsequently, results in immune cells activation, endothelial damage and platelet activation and aggregation. All of these pro-thrombotic responses cumulatively lead to the thrombus formation.

The activated ECs under different circumstances like pathological inflammatory and thrombotic stimuli induces the release of microparticles (MPs). These endothelial-derived MPs are extracellular submicrometer vesicular structure with some RNAs and cytosolic content retained in them (Zwicker et al., 2011). They are shown to be involved in endothelial dysfunction by suppressing the synthesis of NO, prostacyclin and release of TFs (Brodsky et al., 2004; Foley and Conway, 2016). MPs also regulates inflammation, coagulation, adhesion and recruitment of leukocyte. MPs can also be derived from platelets and monocytes with blood borne MPs bearing TF readily detectable in a variety of clinical presentations and might serve as a useful biomarker in identifying patients at the risk of thrombosis (Owens and Mackman, 2011). A study demonstrated that monocytic MPs activated ECs via NLRP3 inflammasome-mediated activation that induced phosphorylation of ERK1/2, activation of the nuclear factor-κB pathway and expression of cell adhesion molecules intercellular adhesion molecule-1, vascular cell adhesion molecule-1, and E-selectin (Wang et al., 2011). Further, NLRP3 activation in ECs has been observed in the endothelial inflammatory responses leading to arterial inflammation and endothelial dysfunction (Xia et al., 2014). In ECs, heme act as pro-inflammatory stimuli which activate NLRP3 inflammasome and subsequent production of IL-1β (Erdei et al., 2018). Besides, thioredoxin (TRX) interacting protein (TXNIP) in ECs, pancreatic islets β cells and monocytes is shown to specifically bind to NLRP3 leading to its activation and release of caspase-1 and IL-1β (Liu et al., 2018). TXNIP is an inhibitor of ROS scavenging protein TRX, which is linked to insulin resistance. The release of IL-1β plays an important role in sterile inflammation by the production of additional pro-inflammatory mediators and upregulation of various adhesion molecules on ECs. Figure 1 schematically depicts inflammasome activation leading to inflammation that subsequently results in endothelial damage, platelet activation and aggregation. All of these pro-thrombotic responses cumulatively lead to the thrombus formation.

Furthermore, employing NLRP3 targeting miRNA might serve as a successful therapeutic approach for the treatment of thrombotic features (Huang et al., 2017; Neudecker et al., 2017). Huang et al. (2017), demonstrated the effect of miR-22 in lowering the levels of pro-inflammatory cytokines by inhibiting the NLRP3 inflammasome pathway, which suppresses coronary arterial ECs apoptosis in rats with coronary heart disease. miRNA regulation of NLRP3 inflammasome is very well documented. NLRP3 regulation by miR-223 (Bauernfeind et al., 2012), miR-223-3p (Boxberger et al., 2019), miR-9 (Wang et al., 2017) has been demonstrated extensively. These miRNAs directly target NLRP3 components, caspase-1 and caspase-8 and downregulate pro-inflammatory cytokines IL-1β and IL-18 (Bauernfeind et al., 2012; Boxberger et al., 2019). One study demonstrated the downregulation of NLRP3 expression, caspase-1 activation and secretion of proinflammatory cytokines IL-1β by increased expression of miR-9 on oxLDL stimulated human primary peripheral blood monocytes and human THP-1 derived macrophages via JAK1/STAT pathway affecting atherosclerosis inflammation (Wang et al., 2017). Such studies suggest that the increased expression of several miRNAs can regulate NLRP3 expression and subsequently potentially abrogate the inflammation and its related diseases such as various inflammatory disorder and thrombosis. Besides NLRP3, NLRP1 is also suggestive of responsible for endothelial dysfunction by regulation of immune-inflammatory processes in arterial ECs (Bleda et al., 2014). Their case-control study having patients with symptomatic peripheral arterial diseases showed that the plasma factors from patients induced NLRP1 expression in ECs suggestive of a new potential target for the therapy.

## Inflammasome in Platelets Activation

Endothelial activation, as discussed above, not only creates a platform for the recruitment and interaction of various immune cells but also aids in the activation of platelets (Yau et al., 2015). Platelets vWF receptor GPIbα is essential for its interaction and recruitment to the endothelial as well as leukocytes and progression of thrombosis (von Brühl et al., 2012). Platelet recruitment also depends on platelet C-type lectin-like receptor 2 (CLEC-2), a platelet membrane molecule capable of binding podoplanin (Inoue, 2017). In its absence, venous thrombosis is suppressed. Under normal condition, the interaction of podoplanin with CLEC-2 is prevented. However, upon activation, platelets secrete many immune mediators, growth factors, chemokines and cytokines that assist its interaction with ECs, leukocytes like monocytes and lymphocytes (Rainger et al., 2015). In addition, three types of granules namely alpha, dense and lysosomes secreted by platelets contribute to its pathogenetic role. Alpha granules store many proteins like platelet factor 4, RANTES (Regulated upon activation, normal T cell expressed and secreted) and β-thromboglobulin, which are important mediators that regulate both inflammation and thrombosis. Dense granules store ATP, ADP, glutamate, polyphosphates, and serotonin. Out of these, serotonin mediates vasoconstriction and vascular permeability. Lysosomes contain enzymes important for protein and matrix degradation like cathepsin, elastase, phosphatase, and glycosidases (Nurden, 2011). Furthermore, platelets are the main source of cyclooxygenase (COX) and its products. COX products thromboxane A2 (TXA2) activates platelets causing vasoconstriction (Schrör, 1993; Belton et al., 2003). Platelets recruitment to the venous wall exposes high-mobility group box 1 (HMGB1), a DNA binding protein, which is released into the extracellular space and acts as a DAMP. It induces the recruitment of leukocytes and new platelets and their activation at the site of thrombus formation as well as secretion of proinflammatory cytokines (Stark et al., 2016).

Pyrin domain containing 3, TLR4, and Bruton tyrosine kinase (BTK) has recently been identified as critical regulators of platelet aggregation and thrombus formation (Vogel and Thein, 2018). BTK, a cytoplasmic tyrosine kinase, acts as a critical regulator of platelet NLRP3 activation. Murthy et al. (2017) observed platelet activation, aggregation and *in vitro* thrombus formation initiated by BTK-dependent platelet NLRP3 inflammasome. All these phenotypes were shown to be decreased by the pharmacological inhibition or genetic ablation of BTK in platelets (Murthy et al., 2017). Vogel et al. (2018) demonstrated NLRP3-dependent increase in platelet caspase-1 activity in sickle cell disease patients (Vogel and Thein, 2018). They also observed the upregulation of HMGB1 and BTK along with NLRP3 that suggest their interplay in caspase-1 activation and platelet aggregation. Further, an elaborative study demonstrated the role of NLRP3 in platelet integrin αIIbβ3 signaling transduction in hemostasis and arterial thrombosis (Qiao et al., 2018). They showed that NLRP3 deficiency significantly decreased platelet spreading on immobilized fibrinogen and impaired clot retraction. The study suggested that the effect of NLRP3 on αIIbβ3 signaling might be through IL-1β as they found significantly reduced IL-1β release from NLRP3-deficient platelets.

## Future Perspectives and Conclusion

A better understanding of inflammation-induced thrombosis could help us in the identification of newer effective therapeutic interventions. For example, some potent and specific NLRP3 inhibitors such as MCC950 might find utilization in the management of this disease. Thus, the investigation of the drugs targeting inflammasome can open an entirely new line of treatment for thrombosis. It has now been proven that activation of NLRP3 under hypoxia potentiates the prothrombotic tendencies (Gupta et al., 2017), but the exact molecular mechanism by which inflammasome exerts its influence in the pathophysiology of thrombosis needs to be explored. The precise signaling pathways through which NLRP3 contributes to endothelial and platelet activation is still ambiguous. Therefore, elucidation of these pathways that are targeted by inflammasome under hypoxia could provide the clue toward the integrated involvement of hypoxia-NLRP3 inflammasome to the underlying mechanisms of vascular dysfunction/hypercoagulation. It will also help in estimating the individual contribution as well as the interaction of the inflammation and coagulation components in the thrombus formation. For example, one can hypothesize regulation of several pathways relevant to vascular homeostasis under low oxygen condition such as TF expression, oxidative stress through activation and signaling of NLRP3. Proper elucidation of the influence of inflammasomes in the activation of these pathways and their implications in the context of thrombosis will help us in the development of their translational applications. Such explanations could be beneficial in prevention of several other cardiovascular and inflammatory disorders that are accompanied by strong thrombotic features.

## Author Contributions

SC and AM contributed equally in the writing and organizing the manuscript. MS edited the manuscript. MA supervised and edited the manuscript.

## Conflict of Interest

The authors declare that the research was conducted in the absence of any commercial or financial relationships that could be construed as a potential conflict of interest.
